# Ethical Decision-Making in Medical Practice: The Role of Moral and Business Philosophies

**DOI:** 10.3390/healthcare13243296

**Published:** 2025-12-15

**Authors:** George Dumitru Constantin, Ruxandra Elena Luca, Ioana Veja, Crisanta-Alina Mazilescu, Bogdan Hoinoiu, Teodora Hoinoiu, Ioana Roxana Munteanu, Roxana Oancea

**Affiliations:** 1 Discipline of Clinical Skills, Department I Nursing, “Victor Babeș” University of Medicine and Pharmacy Timișoara, 300041 Timișoara, Romania; george.constantin@umft.ro (G.D.C.); tstoichitoiu@umft.ro (T.H.); 2 Doctoral School, “Victor Babeș” University of Medicine and Pharmacy Timișoara, 300041 Timișoara, Romania; 3 Center for Advanced Research in Cardiovascular Pathology and Hemostaseology, “Victor Babeș” University of Medicine and Pharmacy Timișoara, 300041 Timișoara, Romania; 4 University Clinic of Oral Rehabilitation and Dental Emergencies, Faculty of Dentistry, “Victor Babeș” University of Medicine and Pharmacy Timișoara, 300041 Timișoara, Romania; luca.ruxandra@umft.ro (R.E.L.); hoinoiu@umft.ro (B.H.); munteanu.roxana@umft.ro (I.R.M.); 5 Interdisciplinary Research Center for Dental Medical Research, Lasers and Innovative Technologies, 300070 Timișoara, Romania; 6 Department of Dental Medicine, Faculty of Dentistry, “Vasile Goldis” Western University of Arad, 310025 Arad, Romania; 7 Teacher Training Department, Politehnica University Timișoara, 300006 Timișoara, Romania; 8 Translational and Experimental Clinical Research Centre in Oral Health, Department of Preventive, Community Dentistry and Oral Health, Faculty of Dental Medicine, “Victor Babeș” University of Medicine and Pharmacy Timișoara, 300041 Timișoara, Romania; roancea@umft.ro

**Keywords:** ethical decision-making, moral philosophy, healthcare professionals, business ethics, legalism, moral pluralism, ethical reasoning profiles, clinical dilemmas

## Abstract

Background: Ethical decision-making in medical care increasingly requires balancing clinical values, professional duties, and organizational reasoning. Understanding how healthcare professionals navigate moral dilemmas necessitates examining the philosophical orientations that shape their ethical judgments. Alongside traditional medical ethics, a business ethics perspective highlights organizational and managerial dimensions of healthcare, offering a more comprehensive understanding of ethical decision-making in modern clinical contexts. Aim: This study aims to examine how healthcare professionals reason about ethical dilemmas by mapping their moral orientations and decision-making patterns across five ethical frameworks-idealism, relativism, objectivism, legalism, and Machiavellianism-integrating both medical and business ethics perspectives. Methods: A cross-sectional study was conducted with 277 participants (medical doctors and students). Two validated instruments were used: the *Attitudes Toward Business Ethics Questionnaire* (ATBEQ) to assess moral orientations and the *Clinical Ethical Dilemmas Questionnaire* (Richeux & Duquéroy) to evaluate ethical decision patterns. Data were analyzed using correlation, multiple regression, and k-means cluster analyses. Results: Among the five orientations, Legalism negatively predicted “It depends” responses (i.e., higher Legalism scores were associated with fewer indecisive responses), indicating greater decisiveness in ethically ambiguous situations. Unexpected positive correlations were also found between traditionally opposing constructs-such as Ethical Relativism and Moral Objectivism-suggesting moral pluralism. The overall regression model was not statistically significant (R^2^ = 0.04, *p* = 0.08). Cluster analysis identified four distinct ethical reasoning profiles: High Machiavellian Idealists, Pragmatic Relativists, Context-Sensitive Objectivists, and Ethical Purists. Conclusions: Abstract philosophical orientations showed limited predictive power for contextual ethical decision-making, highlighting the complex and multidimensional nature of moral reasoning in healthcare. Findings inform the design of context-sensitive ethics education programs that integrate philosophical reflection with case-based learning to strengthen ethical competence among medical professionals.

## 1. Introduction

The medical workforce currently faces increasingly complex challenges arising from demographic shifts, the growing prevalence of chronic diseases, digital transformation, and heightened ethical and legal accountability. Healthcare professionals must not only navigate clinical uncertainty but also uphold ethical standards such as patient autonomy and data confidentiality while managing contextual challenges, including institutional power dynamics, organizational constraints, and resource scarcity. These conditions reflect what has been termed a VUCA environment-volatile, uncertain, complex, and ambiguous [1]-that requires not only technical proficiency but also emotional resilience, strategic adaptability, and sound ethical judgment.

The ability of healthcare providers to operate effectively in such a context is influenced by their ethical decision-making competence, communication skills, and motivational regulation, supported by institutional frameworks and clinical ethicists who facilitate shared moral deliberation. Ethical dilemmas-situations in which competing principles such as autonomy, beneficence, nonmaleficence, and justice conflict-are increasingly common in both clinical and organizational practice [2]. Consequently, modern medical education must cultivate both clinical reasoning and ethical literacy, preparing practitioners to address moral and managerial dimensions of care [3].

In recent years, the gradual inclusion of ethics, management, and leadership modules within some medical curricula has reflected a growing recognition-particularly in European and institutional reform contexts-that sustainable healthcare requires ethically competent and strategically aware professionals [4]. While medical ethics traditionally concerns the doctor–patient relationship and deontological duties, business ethics-the reflection on right and wrong within professional and organizational decision-making-offers a complementary lens for understanding moral reasoning in healthcare institutions [5]. Business ethics is commonly defined as the study of right and wrong, or good and bad, in professional activities and decisions [6]. In healthcare, such reasoning extends beyond individual care to encompass equity, transparency, accountability, and the management of conflicting interests [7]. Medical professionals increasingly act as both clinicians and organizational decision-makers, where moral judgment intersects with administrative, financial, and societal considerations.

While the present study incorporates concepts from business ethics to illuminate organizational accountability, resource management, and decision-making within healthcare institutions, this framing is used in a complementary-not overarching-way. Business ethics serves here as an analytical lens for understanding systemic and managerial dimensions of ethical reasoning, rather than as a universal theoretical authority. Although originating in business contexts, concepts from business ethics-such as accountability, fairness, and transparency-are increasingly relevant to healthcare, where professionals face similar institutional and managerial challenges. In this study, business ethics serves as a complementary framework that helps illuminate how organizational and systemic factors shape clinical moral reasoning.

This cross-domain approach enables the exploration of how managerial reasoning models, inform clinical decision-making, bridging the principles of business ethics with the ethical complexity of medical practice.

Over time, several philosophical orientations have shaped the study of ethical decision-making. Reidenbach and Robin [8] developed the Multidimensional Ethics Scale (MES), which assesses moral equity, relativism, and contractualism. In parallel, Preble and Reichel [9] introduced the Attitudes Toward Business Ethics Questionnaire (ATBEQ), grounded in five ethical orientations commonly discussed in the business ethics literature: Moral Objectivism, Legalism, Machiavellianism, Social Darwinism, and Ethical Relativism [10,11]. These frameworks have since been widely used to examine moral reasoning across professional contexts. Together, these conceptual approaches provide a structured basis for analyzing how individuals interpret and respond to ethical dilemmas across professional contexts. These constructs represent theoretical moral frameworks that have been used to analyze reasoning across professional and cultural contexts, providing conceptual lenses rather than direct determinants of ethical behavior in healthcare practice.

The ATBEQ provides a measure of general ethical orientations, whereas the clinical dilemmas questionnaire captures applied decision-making in real healthcare scenarios. Together, they allow examination of how broader moral beliefs may influence clinicians’ responses when facing context-dependent ethical conflicts.

Building on this tradition, the present study investigates how these moral–philosophical orientations influence ethical decision-making within the healthcare domain. Specifically, it explores how Ethical Relativism, Moral Objectivism, Machiavellianism, Social Darwinism, and Legalism relate to the degree of decisional hesitation in clinical moral dilemmas, expressed through “It depends” responses. Furthermore, the study examines whether distinct moral reasoning profiles emerge from the combination of these orientations and decision tendencies. Beyond moral–philosophical orientations such as relativism or objectivism, ethical decision-making in healthcare is also shaped by legal and institutional contexts. Regulations related to professional accountability or conscience clauses may intensify conflicts between personal convictions and professional duties, influencing clinicians’ moral reasoning. Recent European studies have shown that legal ambiguity regarding end-of-life care and professional autonomy can heighten ethical tension and indecision among healthcare professionals [12].

Therefore, the aim of this study was to analyze the relationships between philosophical orientations and ethical indecision among healthcare professionals and students, and to identify distinct moral reasoning profiles that reflect integrated ethical styles.

Based on previous theoretical and empirical findings, six hypotheses were proposed:

**H1:**  *Higher scores on Ethical Relativism will be associated with a greater proportion of “It depends” responses* [13].

**H2:**  *Higher scores on Moral Objectivism will be associated with fewer “It depends” responses* [14].

**H3:**  *Higher scores on Machiavellianism will be associated with fewer “It depends” responses, reflecting pragmatic and outcome-oriented reasoning* [15].

**H4:**  *Higher scores on Social Darwinism will be associated with fewer “It depends” responses, reflecting competitive or hierarchical pragmatism* [10].

**H5a:**  *In ambiguous or unregulated dilemmas, higher Legalism will predict more “It depends” responses, due to reliance on external norms in unclear contexts* [7].

**H5b:**  *In clearly regulated dilemmas, higher Legalism will predict fewer “It depends” responses, indicating decisiveness where formal rules exist* [7].

**H6:**  *Distinct clusters of ethical reasoning (e.g., Principled Objectivists, Pragmatic Relativists, Legalistic Conformists) will emerge, representing integrated moral reasoning styles among healthcare professionals* [16].

## 2. Materials and Methods

### 2.1. Participants

Participants were eligible for inclusion if they were healthcare professionals or medical students currently engaged in clinical training or practice and had at least one year of professional or academic experience in patient care. Individuals who did not provide informed consent or had incomplete questionnaire responses were excluded. All procedures were conducted in accordance with the Declaration of Helsinki and approved by the Ethics Committee of the Victor Babeș University of Medicine and Pharmacy, Timișoara, Romania (approval No. 87/20.10.2023 rev 2025, 20 October 2023).

A total of 277 participants took part in this study. The sample was predominantly female (77.6%) and composed mainly of practicing physicians (67.9%), followed by medical and dental students (19.5%). Regarding field of specialization, Dentistry accounted for the majority (61.4%), while General Medicine represented 26.4%. Most respondents were in the early or mid-career stages, with 14.8% having no clinical experience, 19.9% having 1–2 years, 26.7% having 3–5 years, and another 26.7% having 5–10 years of experience. Only 4.3% reported over 20 years of professional activity.

Participants were recruited through a convenience sampling strategy, using professional networks, academic institutions, and social media channels. Although this approach facilitated access to diverse healthcare professionals, it may limit the generalizability of findings due to potential self-selection bias and demographic homogeneity.

The participant sample was heterogeneous, including both practicing physicians and medical or dental students. Subgroup characteristics (e.g., professional status, field, and years of experience) were analyzed separately and reported descriptively.

### 2.2. Instruments

Attitudes Toward Business Ethics Questionnaire (ATBEQ)

Moral–philosophical orientations were operationalized across five dimensions: Machiavellianism, Moral Objectivism, Social Darwinism, Ethical Relativism, and Legalism. These were assessed using the Attitudes Toward Business Ethics Questionnaire (ATBEQ) [9], a validated 30-item instrument that presents practical ethical scenarios rated on a five-point Likert scale (1 = strongly disagree, 5 = strongly agree).

Rule adherence was assessed through scenario-based items in the ethical decision-making questionnaire. Participants indicating consistent reliance on codified principles-such as institutional regulations, professional standards, or legal norms-were classified as displaying higher rule-based consistency (Legalism). For example, selecting options emphasizing ‘compliance with professional codes’ or ‘following established procedures’ in multiple dilemmas indicated a strong legalistic orientation.

The study draws on five established schools of moral thought: Idealism (the belief that morally right actions should avoid causing harm and promote the welfare of others, without implying a utilitarian requirement to maximize overall outcomes), Relativism (emphasizing context and cultural norms), Objectivism (belief in universal moral principles), Legalism (rule-based ethical consistency), and Machiavellianism (strategic or outcome-oriented moral reasoning). These orientations provide a multidimensional basis for analyzing ethical decision-making among healthcare professionals.

For this study, participants were instructed to consider each item from the standpoint of their medical or dental practice. Although this adaptation ensured contextual relevance, it represents a limitation, as no formal validation of the adapted version was conducted. Items were, however, reviewed by two academic experts in medical ethics for linguistic and conceptual clarity. The instrument demonstrated good internal reliability in this sample (Cronbach’s α = 0.81). Future studies should formally validate this contextual adaptation to strengthen psychometric equivalence. Due to copyright restrictions, the full instruments cannot be reproduced in the manuscript. However, all items were reviewed by two academic experts in medical ethics to ensure linguistic clarity and clinical relevance. Only minor wording adjustments were made to contextualize the scenarios for healthcare professionals, while preserving the original structure, scoring format, and psychometric intent of the questionnaires. Further details regarding the adaptations are available from the authors upon reasonable request.

**Clinical Ethical Dilemmas Questionnaire (Richeux & Duquéroy)**

Ethical decision tendencies were evaluated using the Clinical Ethical Dilemmas Questionnaire developed by Richeux and Duquéroy [17]. The instrument comprises 35 clinical scenarios addressing autonomy, confidentiality, and professional conduct dilemmas, each offering three possible responses: Yes, No, and It depends. The latter option (It depends) was interpreted as reflecting ethical indecision or contextual moral hesitation, consistent with previous literature on moral complexity and ambivalence [13,14].

The study design combined two validated questionnaires administered jointly to the same participants. The first measured moral orientations (Idealism, Relativism, Objectivism, Legalism, Machiavellianism), while the second assessed decision-making responses to clinical ethical dilemmas. This mixed design enabled the identification of relationships between moral-philosophical profiles and situational ethical choices, addressing the main research objective of linking abstract moral reasoning to applied clinical decision-making.

### 2.3. Data Collection and Ethical Considerations

Data were collected via an online structured questionnaire distributed over a three-month period. Participants were invited through institutional mailing lists, professional organizations, and social media groups. Two reminder messages were issued to enhance participation. Respondents received detailed instructions regarding data confidentiality and study purpose.

Informed consent was obtained electronically from all participants prior to inclusion. They were assured of the voluntary nature of participation and the anonymity of responses. The study was approved by the Ethics Committee of the “Victor Babeș” University of Medicine and Pharmacy, Timișoara, Romania (Protocol No. 87/20.10.2023 rev. 2025).

### 2.4. Data Analyses

Descriptive statistics (means, standard deviations, frequencies) were computed for all variables. The It_Depends responses were aggregated as the percentage of indecisive answers per participant, providing a continuous measure of ethical indecision. Histogram inspection confirmed approximate normality.

To explore associations among the five moral–philosophical dimensions (Machiavellianism, Ethical Relativism, Moral Objectivism, Legalism, and Social Darwinism) and situational indecision (It_Depends), both Pearson’s and Spearman’s correlation analyses were conducted to ensure robustness against potential non-normality.

A multiple linear regression analysis was then performed using It_Depends as the dependent variable and the five moral–philosophical dimensions as predictors (H1–H5a and b). Model diagnostics included examination of residual plots, Variance Inflation Factors (VIF < 2.0), and tolerance statistics to assess multicollinearity.

Finally, an exploratory k-means cluster analysis (H6) was applied to identify distinct ethical reasoning profiles based on participants’ standardized scores across the five philosophical dimensions and the It_Depends variable. The number of clusters was determined through evaluation of within-group variance reduction and silhouette coefficients, ensuring conceptual interpretability and empirical stability of the resulting profiles.

Data from Table 1 provided standardized mean scores for each moral orientation, which were then used as predictors in correlation and regression analyses (Table 2) and as input variables in cluster analysis (Table 3). This procedure allowed us to determine how specific moral orientations-such as Machiavellianism and Legalism-were associated with decisiveness, ethical consistency, and distinct reasoning profiles among healthcare professionals.

## 3. Results

### 3.1. Level of Indecision and Philosophical Orientations in Clinical Decision-Making

Analysis focused on the “It depends” responses recorded in the Clinical Ethical Dilemmas Questionnaire, as these indicate internal tension between competing moral principles that hinder participants from adopting a definitive “Yes” or “No” stance. Prior research associates such undecided responses with moral complexity and cognitive deliberation, reflecting simultaneous consideration of ethical values, professional norms, and contextual constraints [2,16].

By examining “It depends” responses, this study aimed to capture nuanced forms of ethical uncertainty, revealing situations in which no moral principle could be applied exclusively without violating another of comparable weight. These correspond to what Kidder [18] termed “pure ethical dilemmas”, in which each possible choice entails both moral benefits and ethical risks.

From an educational perspective, this focus offers practical insight into areas where ethical hesitation is most pronounced, helping to identify priorities for targeted ethics training in clinical practice.

#### “It Depends” Responses

The highest frequencies of “It depends” responses were observed in dilemmas concerning treatment refusal, inter-professional accountability, and end-of-life decisions-contexts characterized by high emotional, legal, and relational complexity (Table 1). Such scenarios typically involve conflict between autonomy, beneficence, and nonmaleficence, explaining the elevated indecision rates.

### 3.2. Correlation Analyses

A Pearson correlation analysis examined associations among It_Depends and the five moral–philosophical dimensions (Table 3). Moderate positive correlations were found among most orientations, suggesting interconnected rather than mutually exclusive ethical frameworks.

Machiavellianism exhibited the strongest positive correlations with other moral constructs (r = 0.38–0.45, *p* < 0.001), indicating its central integrative role within moral reasoning. Ethical Relativism also correlated positively with Moral Objectivism (r = 0.28, *p* < 0.001) and Legalism (r = 0.30, *p* < 0.001), suggesting that participants often combine flexible and principle-driven approaches rather than adhering to a single ethical orientation.

A complementary Spearman’s rank correlation analysis yielded an equivalent pattern of associations (see Table 4), confirming the robustness and consistency of the observed relationships across both parametric and nonparametric methods.

These findings collectively indicate that moral reasoning among healthcare professionals is multidimensional and overlapping, challenging traditional assumptions that ethical orientations operate in isolation.

The Spearman analysis confirmed the robustness of the relationships observed in the Pearson matrix. Machiavellianism again emerged as the most strongly interconnected construct, followed by Ethical Relativism and Idealism–Legalism. These consistent patterns suggest a stable multidimensional structure of moral reasoning, where different ethical orientations coexist rather than compete.

### 3.3. Multiple Linear Regression Analysis

A multiple regression model assessed whether the five philosophical orientations predicted ethical indecision (It_Depends).

The overall model was not statistically significant, F(5, 271) = 1.99, *p* = 0.08, R^2^ = 0.04, indicating that the predictors jointly explained only 4% of the variance in situational decision-making. Diagnostic checks confirmed no multicollinearity (VIF range = 1.21–1.46; tolerance > 0.68).

Only Idealism/Legalism emerged as a significant negative predictor (B = −0.69, SE = 0.30, β = −0.15, 95% CI [−1.28, −0.11], *p* = 0.021), indicating that participants with higher rule-based or idealistic orientations were less likely to choose “It depends,” thus showing greater decisiveness. The remaining variables were non-significant (*p* > 0.10).

These findings suggest that rule adherence promotes decisional clarity, whereas relativistic or pragmatic orientations do not necessarily translate into ethical hesitation.

### 3.4. Cluster Analysis (K-Means)

An exploratory k-means cluster analysis (k = 4, silhouette = 0.71) identified four distinct ethical reasoning profiles, reflecting diverse ways of integrating philosophical orientations into decision-making patterns. ANOVA confirmed significant between-cluster differences for all six variables (*p* < 0.001).

Cluster 1: High Machiavellian Idealists (*n* = 52)-High across all moral dimensions, combining strategic pragmatism with idealistic rule orientation. This hybrid pattern reflects moral integration rather than contradiction, consistent with multidimensional moral cognition theories [19,20].

Cluster 2: Pragmatic Relativists (*n* = 72)-Moderate-to-high Machiavellianism and Ethical Relativism, low Legalism, and the highest It_Depends rates. Their reasoning is context-sensitive and situationally adaptive, resembling Forsyth’s “situationist” type [21].

Cluster 3: Context-Sensitive Objectivists (*n* = 69)-Moderate scores across orientations and the lowest indecision levels, reflecting principled consistency with contextual awareness [22].

Cluster 4: Ethical Purists (*n* = 84)-Uniformly low moral orientation scores but moderate-to-high It_Depends rates, indicating ethical sensitivity without conceptual integration, a profile associated with limited moral schema development [23].

### 3.5. Linear Discriminant Analysis (LDA)

To validate the cluster structure, an LDA was conducted. Three discriminant functions explained 72.0%, 22.8%, and 5.2% of the between-cluster variance, respectively. The overall classification accuracy was 76.4%, indicating robust differentiation. The cluster analysis should be interpreted as a tentative, exploratory approach, given the minimal explained variance and weak inter-variable associations. Its purpose is to generate preliminary hypotheses about potential ethical reasoning profiles rather than to support definitive conclusions.

- **LD1** separated clusters based on overall moral–philosophical orientation (|*r*| ≥ 0.55 for all dimensions).
- **LD2** differentiated participants primarily by decisional tendency (It_Depends, *r* = 0.82).
- **LD3** reflected a continuum between Ethical Relativism (*r* = 0.57) and Legalism (*r* = −0.48), distinguishing flexible versus rule-based reasoning styles.

Collectively, these results confirm the presence of four stable and interpretable moral reasoning profiles, underscoring the heterogeneous and multidimensional nature of ethical decision-making among healthcare professionals.

## 4. Discussion

The present study examined how healthcare professionals reason about ethical dilemmas by linking moral–philosophical orientations with situational decision-making. A considerable proportion of dilemmas reported by participants involved disregard of patient wishes and professional disagreements, reflecting the relational and systemic tensions that persist in many clinical environments. These results highlight not only the ethical complexity of end-of-life care and therapeutic futility, but also the intricate interpersonal dynamics that shape moral reasoning in healthcare.

Consistent with international findings, the most frequently reported ethical challenges were related to treatment refusal, inter-professional accountability, and end-of-life decisions, particularly in cases where family preferences were disregarded or withdrawal of life-sustaining treatment was discussed. Comparable concerns have been observed in other healthcare systems, where uncertainty surrounding legal and professional responsibilities generates moral distress and ethical indecision [12]. These parallels suggest that ethical uncertainty often arises at the intersection of professional duty, family expectations, and legal frameworks. Although these issues appear especially significant, other dilemmas-such as boundary violations or failures in communication-may exert subtler but equally meaningful ethical consequences.

The regression model explained only a small proportion of variance (R^2^ = 0.04), indicating that classical moral constructs account for a limited part of moral behavior in real clinical contexts. Given the small effect sizes and limited proportion of variance explained by the regression model, the present findings should be interpreted as exploratory. They offer initial insights into possible relationships between general ethical orientations and contextual decision-making, which require further investigation in larger and more diverse samples. This aligns with previous research suggesting that abstract ethical orientations, while theoretically valuable, have limited predictive power in practical situations [19,20,21]. Nevertheless, the Idealism–Legalism dimension emerged as a significant predictor of decisiveness. Participants with stronger rule-based and idealistic orientations gave fewer “It depends” responses, indicating that adherence to principles provides moral clarity under conditions of uncertainty. Clinically, these professionals tend to rely on codified norms-such as institutional protocols or professional codes-to ensure fairness and consistency in sensitive decisions, including those concerning informed consent and end-of-life care.

Recent studies emphasize that legal ambiguity may further influence ethical decision-making. For instance, unclear regulations-such as the interpretation of conscience clauses in end-of-life situations-may generate conflict between professional obligations and personal moral convictions [12]. This suggests that rule-based reasoning interacts not only with moral principles but also with the clarity of legal and institutional frameworks.

Contrary to initial expectations, Ethical Relativism did not predict indecision. This finding suggests that contextual sensitivity does not necessarily lead to hesitation; rather, it may coexist with confidence in moral judgment once professional norms are internalized. Previous research supports this interpretation, viewing relativism as a flexible cognitive stance rather than a source of moral uncertainty [24].

The correlation analysis showed notable overlap among moral orientations, with Machiavellianism emerging as a central integrative dimension. In this context, Machiavellianism does not represent amoral pragmatism but rather strategic adaptability that can coexist with principled reasoning [25,26,27]. The positive association between Ethical Relativism and Moral Objectivism (r = 0.28, *p* < 0.001) further supports the idea that healthcare professionals may simultaneously endorse flexible and universalist perspectives-a hallmark of moral pluralism [11,28]. Such pluralism reflects a sophisticated capacity to adapt ethical reasoning to clinical realities without abandoning core moral principles.

The absence of strong associations between “It depends” responses and any single moral orientation reinforces the view that ethical indecision arises less from philosophical stance and more from contextual or emotional complexity. This perspective is consistent with models that emphasize the interplay of intuition, affect, and situational awareness in moral judgment [29,30].

Cluster analysis added depth to these findings by identifying four distinct ethical reasoning profiles. The first group-highly idealistic yet strategically oriented-combined cognitive flexibility with principled reasoning, reflecting an integrative form of moral thinking. The second cluster showed greater adaptability and situational awareness, aligning with a more pragmatic and context-dependent style. The third cluster displayed moral stability and low indecision, typical of rule-based reasoning. Finally, the fourth group showed moderate indecision with limited integration of moral concepts, suggesting awareness without a consistent cognitive framework.

Linear discriminant analysis confirmed these patterns, with Machiavellianism and Ethical Relativism as the strongest discriminant variables, supporting the idea that these constructs define key dimensions of moral cognition [26,27]. Collectively, these results depict ethical reasoning in healthcare as multidimensional and dynamic, in which professionals balance universal principles, contextual realities, and institutional expectations rather than adhering strictly to a single moral code.

Integrating dual-process and social-intuitionist perspectives, these findings indicate that moral behavior in healthcare arises from the interaction of reflective and intuitive reasoning systems. The coexistence of relativistic and objectivist tendencies exemplifies moral pluralism-a pragmatic synthesis between principle-based and context-sensitive reasoning that more accurately mirrors clinical realities. Healthcare ethics, therefore, should be understood as an evolving interplay between professional duty, situational judgment, and institutional norms.

From a clinical standpoint, these reasoning profiles have direct implications. Practitioners with legalistic–idealistic tendencies may prioritize consistency, compliance, and procedural safety, while more pragmatic individuals may better adapt to diverse patient needs but risk moral ambivalence. Recognizing these tendencies can inform ethics education, interdisciplinary collaboration, and institutional policy, supporting a balance between principled rigor and contextual empathy.

Clinicians may not always articulate the philosophical reasoning behind their actions, yet they are highly aware of the consequences of their ethical choices. This underscores the importance of structured institutional support-such as clinical ethics committees-that can offer independent guidance and facilitate reflective dialogue when uncertainty arises. Expanding such mechanisms would help translate ethical principles into consistent and compassionate clinical practice.

Younger professionals may find it challenging to apply theoretical ethics to practical dilemmas, even when they recognize issues such as futile treatment or inadequate consent procedures. This gap highlights the importance of continuous ethics education and reflective training, particularly regarding voluntary informed consent, which remains one of the most frequently overlooked ethical requirements in both clinical care and research.

Although ethical reflection is a core professional responsibility, many clinicians lack the time and organizational resources to engage in deep moral deliberation. Heavy workloads and decision fatigue can contribute to neglecting fundamental principles such as primum non nocere. Institutions should therefore ensure access to ethics consultation services and dedicated spaces for reflection, integrating them into daily medical practice.

Clinical ethics committees play a crucial role in supporting ethical decision-making, and expanding their presence across healthcare systems would help embed moral principles more effectively into clinical routines. By linking philosophical orientations with real-world ethical reasoning, this study provides a framework that integrates moral philosophy, organizational ethics, and practical decision-making, extending prior research on professional moral reasoning [31,32,33].

Overall, these findings reinforce that classical moral frameworks explain only a small part of ethical behavior in clinical contexts (R^2^ = 0.04), supporting models in which emotion, intuition, and context shape moral judgment [11,19,20,21,28,29,30]. The identified profiles, particularly those combining principled and strategic reasoning, illustrate that ethical decision-making is neither purely rational nor entirely situational but arises from the integration of both.

The study also acknowledges several limitations. The sample was predominantly female, highly educated, and culturally homogeneous, limiting the generalizability of results. Self-reported data may have been influenced by social desirability bias and common method variance. The modest explained variance indicates that additional variables-such as emotional regulation, professional experience, or cognitive style-likely play a stronger role [11,28,29,30]. Furthermore, while the questionnaires were linguistically adapted and pilot-tested, the absence of formal psychometric validation may have affected measurement precision. These constraints should be considered when interpreting the associations between moral orientations and ethical decision-making. The use of convenience sampling via institutional networks and open social media groups may have introduced self-selection bias and limited the representativeness of the sample, which should be considered when generalizing the findings.

Future research should extend these findings using cross-cultural and longitudinal designs to explore how institutional and cultural contexts influence moral reasoning. Behavioral and neuroethical methods could provide further insight into the emotional and cognitive mechanisms underlying ethical choices. Larger and more diverse samples are needed to validate these results and to explore how ethical reasoning evolves across professional experience levels and healthcare environments.

## 5. Conclusions

This study examined the relationship between philosophical orientations and indecision in ethical dilemmas, revealing that moral reasoning in healthcare is inherently multidimensional and context-dependent. Although Idealism—Legalism predicted greater decisiveness, the overall model explained only a small proportion of variance, suggesting that situational, emotional, and experiential factors strongly shape ethical decision-making beyond abstract philosophical orientations.

The positive correlations between opposing constructs, such as Ethical Relativism and Moral Objectivism, highlight the coexistence of multiple ethical frameworks within individuals, supporting moral pluralism rather than rigid dichotomies. This complexity reflects how clinicians balance universal principles with contextual realities in everyday care.

The identification of four distinct reasoning profiles-High Machiavellian Idealists, Pragmatic Relativists, Context-Sensitive Objectivists, and Ethical Purists—illustrates diverse moral cognition styles that integrate rule-based, pragmatic, and reflective elements. These profiles extend existing typologies and provide a nuanced understanding of how professionals navigate competing ethical demands.

Practically, these findings underscore the need for ethics education and organizational training that move beyond uniform instruction. Case-based reflection, context-sensitive frameworks, and tailored learning interventions can strengthen ethical competence and decision-making confidence among healthcare professionals.

Ultimately, recognizing moral reasoning as pluralistic, adaptive, and embedded in clinical reality offers a foundation for cultivating ethical awareness that supports both professional integrity and patient trust-cornerstones of clinical excellence in modern healthcare.

## Figures and Tables

**Table 1:** Ethical dilemmas with the highest proportion of *“It depends”* responses.

Ethical Dilemma	% “It Depends”
2.2. Non-respect of family’s treatment wishes	40.79%
2.15. Complaint about another doctor	40.07%
2.26. Favoring a younger patient	38.63%
2.8. Decision to stop treatment	38.27%
2.1. Patient consent	37.18%
2.31. Favoritism toward family in treatment	34.30%
2.3. Family request for nondisclosure	33.57%
2.19. Romantic relationship with a patient	32.85%
2.11. Concealing diagnosis	32.13%
2.28. Professional boundary violation	31.77%
2.33. Abandonment during medical crisis	30.69%
2.9. Treatment without hope of recovery	30.32%

**Table 2:** Summary statistics for moral–philosophical variables.

Variable	*M*	*SD*	*n*	*SE_M_*	Min	Max	Skewness	Kurtosis
Machiavellianism	2.60	0.49	277	0.03	1.00	4.33	0.46	1.11
Ethical Relativism	2.48	0.57	277	0.03	1.00	3.67	−0.20	−0.50
Moral Objectivism	3.21	0.54	277	0.03	1.00	4.67	−0.38	1.46
Idealism/Legalism	2.68	1.17	277	0.07	1.00	5.00	0.30	−0.88
Social Darwinism	2.32	0.47	277	0.03	1.00	3.88	0.08	0.51

**Table 3:** Pearson correlation matrix among It_Depends and moral–philosophical dimensions.

Variable	1	2	3	4	5	6
1. It_Depends	-					
2. Machiavellianism	0.01	-				
3. Ethical Relativism	−0.09	0.45 *	-			
4. Moral Objectivism	−0.07	0.38 *	0.28 *	-		
5. Legalism	−0.14	0.41 *	0.30 *	0.25 *	-	
6. Social Darwinism	0.02	0.43 *	0.30 *	0.28 *	0.22 *	-

Note: * *p* < 0.05.

**Table 4:** Spearman correlation matrix among It Depends, Machiavellianism, Ethical Relativism, Moral Objectivism, Idealism/Legalism, and Social Darwinism.

Variable	1	2	3	4	5	6
1. It_Depends	-					
2. Machiavellianism	0.01	-				
3. Ethical Relativism	−0.1	0.44 *	-			
4. Moral Objectivism	−0.1	0.33 *	0.25 *	-		
5. Idealism/Legalism	−0.12	0.38 *	0.29 *	0.27 *	-	
6. Social Darwinism	0.01	0.36 *	0.26 *	0.22 *	0.19 *	-

Note: * *p* < 0.05.

## Data Availability

The data presented in this study are available on request from the corresponding author. The data are not publicly available due to privacy or ethical restrictions.
